# Dual Enhancement of Optoelectronic and Mechanical Performance in Perovskite Solar Cells Enabled by Nanoplate-Structured FTO Interfaces

**DOI:** 10.3390/nano15181430

**Published:** 2025-09-18

**Authors:** Ruichen Tian, Aldrin D. Calderon, Quanrong Fang, Xiaoyu Liu

**Affiliations:** 1 School of Mechanical, Manufacturing and Energy Engineering, Mapua University, Muralla Street, Intramuros, Manila 1004, Philippines; adcalderon@mapua.edu.ph (A.D.C.); xliu@mymail.mapua.edu.ph (X.L.); 2 School of Graduate Studies, Mapua University, Muralla Street, Intramuros, Manila 1004, Philippines; 3 School of Equipment Maintenance, Hunan Defense Industry Polytechnic, No. 9, Xueshi Road, Yuhu District, Xiangtan 411100, China; 4 School of Computer Science and Technology, Wuhan University, Wuhan 430072, China; quanrong.fang@zju.edu.cn; 5 School of Computer Science and Technology, Zhejiang University, Hangzhou 310058, China

**Keywords:** integrated photovoltaic–storage systems, transparent conducting oxide engineering, flexible energy conversion devices, interfacial mechanical reinforcement, light-management nanostructures

## Abstract

Perovskite solar cells (PSCs) rarely report, on a single-device platform, concurrent gains in optoelectronic efficiency and buried-interface mechanical robustness—two prerequisites for flexible and roll-to-roll (R2R) integration. We engineered a nanoplate-structured fluorine-doped tin oxide (NP-FTO) front electrode that couples light management with three-dimensional interfacial anchoring, and we quantified both photovoltaic (PV) and nanomechanical metrics on the same device stack. Relative to planar FTO, the NP-FTO PSCs achieved PCE of up to 25.65%, with simultaneous improvements in Voc (to 1.196 V), Jsc (up to 26.35 mA cm^−2^), and FF (to 82.65%). Nanoindentation revealed a ~28% increase in reduced modulus and >70% higher hardness, accompanied by a ~32% reduction in maximum indentation depth, indicating enhanced load-bearing capacity consistent with the observed FF gains. The low-temperature, solution-compatible NP-FTO interface is amenable to R2R manufacturing and flexible substrates, offering a unified route to bridge high PCE with reinforced interfacial mechanics toward integration-ready perovskite modules.

## 1. Introduction

The global transition toward sustainable energy has accelerated the development of next-generation photovoltaic (PV) technologies. Among these, perovskite solar cells (PSCs) based on organic–inorganic halide materials have emerged as one of the most promising options due to their outstanding optoelectronic characteristics, including variable bandgaps, long carrier diffusion lengths, high absorption coefficients, and remarkable defect tolerance [1,2,3]. These properties allow PSCs to reach high power conversion efficiencies (PCEs) with relatively thin active layers, thereby reducing material usage and simplifying device architectures [4,5]. Beyond their excellent light-harvesting capability, PSCs offer notable advantages in manufacturability. Unlike conventional silicon-based solar cells that require high-temperature and vacuum-based processes, PSCs can be fabricated using low-temperature, solution-processed techniques compatible with scalable roll-to-roll printing, making them attractive for flexible and building-integrated photovoltaics (BIPV) [6,7]. The certified PCE of PSCs has surged from 3.8% to over 26% in just over a decade, representing the fastest efficiency gain among all solar technologies [4,8,9]. Recent reviews, such as that by Tayari et al. [10], have highlighted these advances while stressing that device stability, interfacial optimization, and scalable processing remain critical barriers for commercial deployment.

In a typical n–i–p-structured PSC, a transparent conducting oxide (TCO), most commonly fluorine-doped tin oxide (FTO), is followed by an electron transport layer (ETL), the perovskite absorber, a hole transport layer (HTL), and a metallic back electrode [10]. Despite considerable progress in material development and device engineering, interfacial losses and optical inefficiencies at the TCO interface remain major bottlenecks to further improvements in efficiency and operational stability [11]. Planar FTO surfaces often cause undesirable optical reflection and non-uniform perovskite nucleation, which compromise both light management and charge extraction [12,13,14]. Moreover, translating laboratory-scale efficiencies into outdoor operation is still challenging [7,15,16]. A key factor is the angular dependence of sunlight incidence, which exacerbates reflection losses in planar architectures and leads to discrepancies between certified and practical device performance [17,18,19]. Non-radiative recombination at the TCO/ETL interface further limits efficiency due to poor energy-level alignment, inadequate passivation, and unfavorable surface morphology in conventional FTO substrates [16,18,19].

Nanostructured TCOs have emerged as an effective strategy to address these issues. In particular, nanoplate-enhanced FTO (NP-FTO) has been proposed to improve both optical and interfacial properties [20,21]. Vertically aligned nanoplates increase light scattering, facilitate heterogeneous perovskite crystallization, and improve interfacial contact, thereby enhancing Jsc, Voc, fill factor (FF), and overall PCE. Prior studies, including our own, have demonstrated that PSCs fabricated on NP-FTO substrates consistently outperform those on flat FTO controls [21,22]. Alongside optoelectronic efficiency, however, mechanical reliability has become increasingly important, especially for flexible, wearable, and BIPV applications. Due to their hybrid composition and multilayer architecture, PSCs are prone to interfacial delamination, fracture, and stress-induced failure [23]. Nanoindentation offers a powerful means to probe interfacial mechanical properties at the nanoscale, providing quantitative measures of hardness, elastic modulus, and interfacial adhesion with high spatial resolution [19,22]. Previous studies have suggested that perovskite morphology and substrate texture influence mechanical stability, but the broader impact of TCO nanostructuring on the mechanical integrity of complete PSC devices remains insufficiently explored [23]. Recent work on sol–gel-derived oxide perovskite frameworks has further emphasized the strong link between structural design, electrical functionality, and mechanical reinforcement, highlighting opportunities for multifunctional interface engineering [24,25].

In this work, we address this gap by systematically investigating the dual effect of NP-FTO on both photovoltaic performance and mechanical robustness. By combining current–voltage characterization with nanoindentation analysis, we demonstrate that NP-FTO simultaneously enhances light harvesting, suppresses non-radiative recombination, and strengthens buried interfaces against stress-induced degradation. The results establish nanoplate-engineered FTO as a scalable, low-temperature, and solution-compatible approach that unifies high efficiency with mechanical durability, thereby advancing PSCs toward integration in flexible and roll-to-roll production platforms [22,26,27].

## 2. Methods

### 2.1. Materials

Nippon Sheet Glass supplied the fluorine-doped tin oxide (FTO) glass substrates, which had a sheet resistance of roughly 15 Ω/sq. For use as the electron transport layer (ETL), a 15 weight percent tin (IV) oxide (SnO_2_) colloidal dispersion was diluted with a hydrogen peroxide and deionized water mixture (volume ratio 1:1:4). Then, 691.5 mg of PbI_2_, 1.5 mg of Pb(SCN)_2_, and 5 mg of CsCl were dissolved in 1 mL of a DMF/DMSO (9:1 *v*/*v*) solvent combination, and the mixture was stirred at 70 °C for 4 h to create the PbI_2_ precursor solution (1.5 M). By dissolving 90 mg of formamidinium iodide (FAI) and 12 mg of methylammonium chloride (MACl) in 1 mL of isopropanol (IPA), and stirring at room temperature for 4 h, the organic ammonium salt solution was produced. Next, 72.3 mg of spiro-OMeTAD was dissolved in 1 mL of chlorobenzene to create the hole transport layer (HTL) solution. Then, 28.8 μL of tert-butylpyridine (TBP) and 17.5 μL of Li-TFSI solution (520 mg/mL in acetonitrile) were added.

Every reagent was used exactly as supplied, requiring no additional purification.

### 2.2. Device Fabrication

FTO substrates were cleaned sequentially with deionized water, acetone, and ethanol by ultrasonic treatment for 30 min each. After drying with nitrogen gas, the substrates were treated with ultraviolet-ozone for 15 min. The SnO_2_/ETL was deposited by spin-coating the diluted colloidal solution at 4000 rpm for 30 s, followed by annealing at 150 °C for 30 min in ambient air.

After cooling, the PbI_2_ precursor solution was spin-coated at 1500 rpm for 30 s and annealed at 70 °C for 1 min in a nitrogen-filled glovebox. Subsequently, the organic salt solution was spin-coated at 2000 rpm for 30 s and annealed at 145 °C for 15 min under ambient conditions (relative humidity 30–40%) to complete the perovskite layer formation.

**Figure 1:**
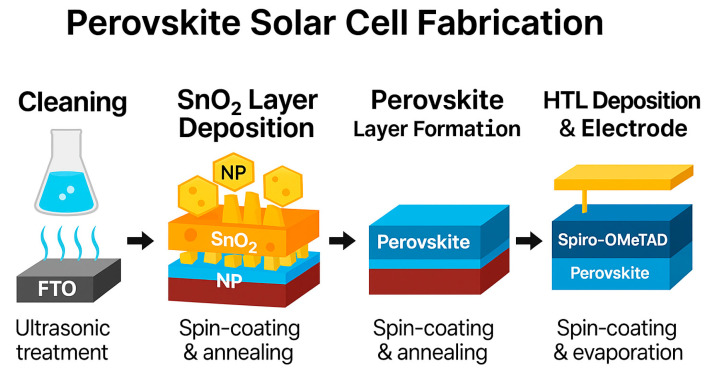
Fabrication flow of perovskite solar cell with layered structure and process parameters.

The HTL layer was deposited by spin-coating the spiro-OMeTAD solution at 3000 rpm for 30 s. Finally, a 70 nm-thick gold electrode was thermally evaporated under high vacuum (~10^−6^ Torr) to complete the device architecture (Figure 1). The active area of each device was defined as 0.0529 cm^2^ [22].

### 2.3. J–V Measurement Procedure

Using a source-measure unit (SMU, Keithley 2400) and standard AM 1.5G illumination (100 mWcm^−2^) supplied by a calibrated solar simulator (Newport, Class AAA), the current density–voltage (J–V) characteristics of the PSCs were measured. A metal shadow mask, usually measuring between 0.1 and 0.16 cm^2^, was used to define the active area of each device, and a certified Si reference cell (Newport) was used to calibrate the light intensity.

All J–V curves were recorded in a quasi-steady-state mode with a voltage scan range from 1.5 V to −0.1 V at a fixed scan rate (typically 100 mV/s) without bias preconditioning. The measurement was conducted under ambient laboratory conditions (temperature ~25 °C, relative humidity <40%), and no device encapsulation was applied during testing.

### 2.4. Nanoindentation Characterization

**Figure 2:**
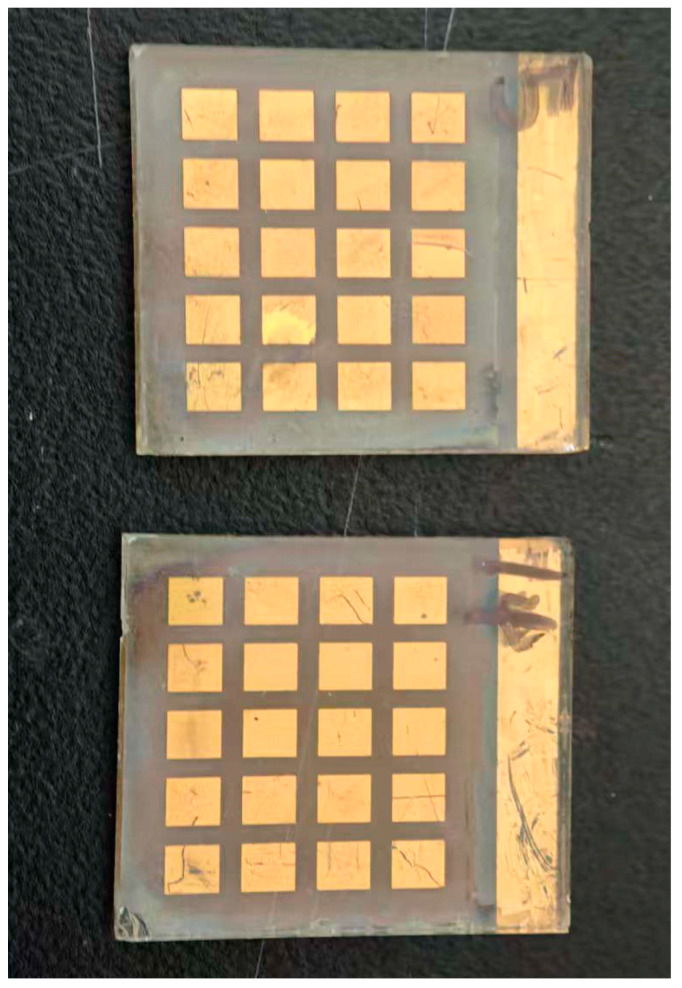
Photograph of the fabricated perovskite solar-cell arrays on normal FTO (**top**) and NP-FTO (**bottom**) substrates.

**Figure 3:**
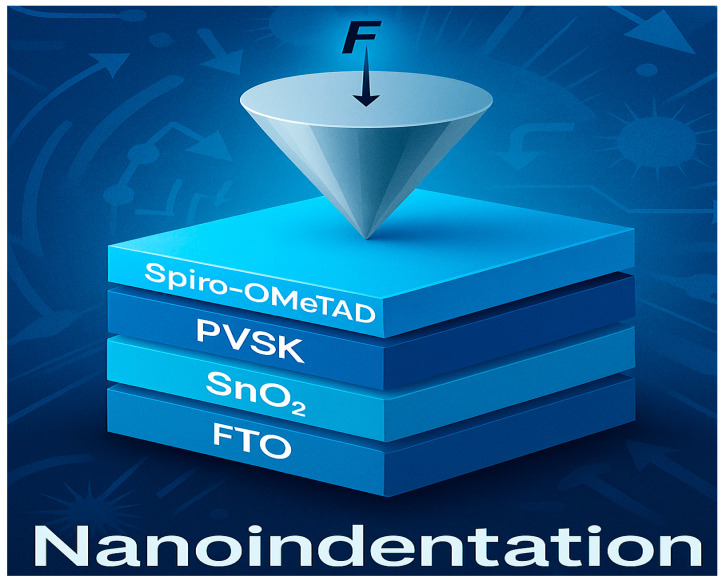
Schematic illustration of nanoindentation on a perovskite solar-cell stack.

Nanoindentation measurements were conducted using a Hysitron TS 77 nanoindenter (Bruker, Ettlingen, Germany), which offers high-precision mechanical characterization at the nanoscale. The instrument features a displacement resolution of 0.01 nm, a time resolution of 0.005 s, and a load resolution of 3 nN, ensuring accurate evaluation of interfacial mechanical properties in multilayer solar cell architectures (Figure 2 and Figure 3).

All measurements were performed using a Berkovich diamond indenter under controlled ambient conditions (temperature ~25 °C, relative humidity <40%). The load–displacement data were acquired in quasi-static mode, with a maximum load of 8000 μN, a loading/unloading rate of 100 μN/s, and a dwell time of 10 s at peak load to minimize viscoelastic effects [7,24].

Indentations were made directly on the perovskite layer surface to assess the mechanical response influenced by the underlying TCO structure (normal FTO vs. NP-FTO). Five indents per sample were performed to ensure statistical reproducibility. The reduced elastic modulus (Er) and hardness (H) were calculated using the Oliver–Pharr method based on the unloading curves. Special care was taken to avoid cracking, substrate effects, and surface artifacts during interpretation [7,25].

## 3. Results and Discussion

### 3.1. Photovoltaic Performance Enhancement via Nanoplate-Structured FTO

**Figure 4:**
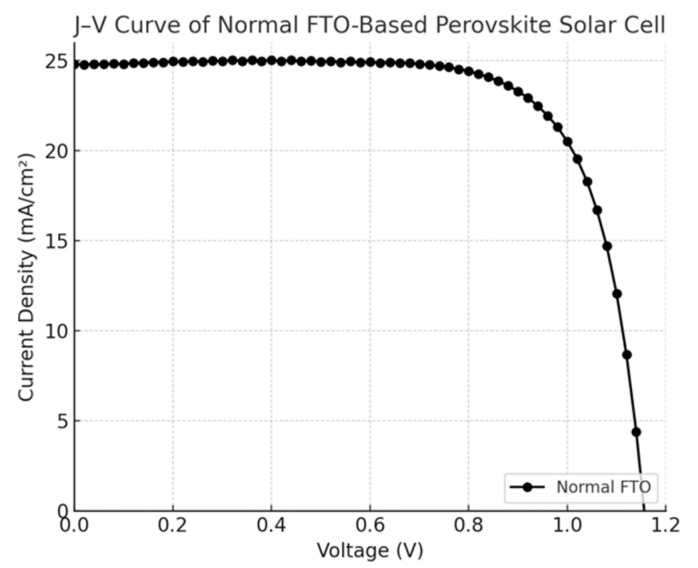
J–V curve of the normal FTO device.

**Figure 5:**
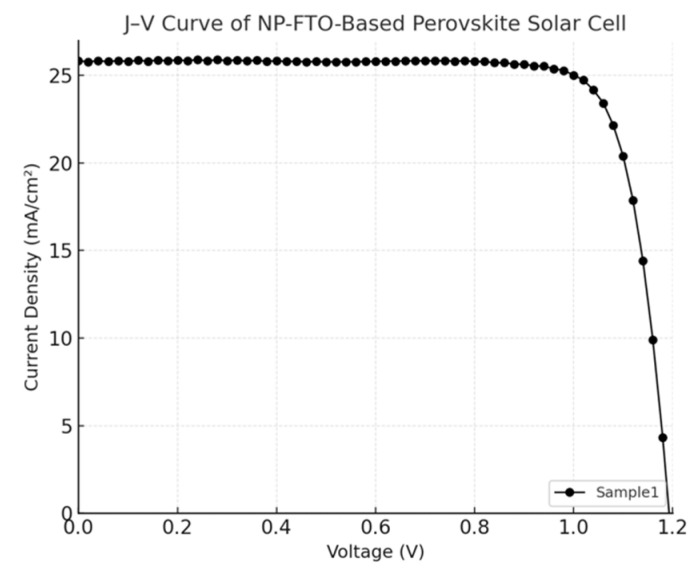
J–V curve of the NP-FTO-Sample1 device.

**Figure 6:**
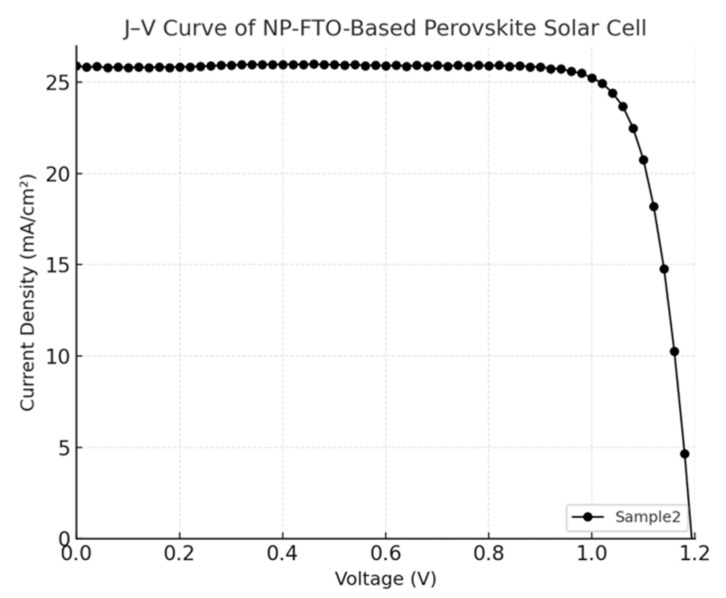
J–V curve of the NP-FTO-Sample2 device.

**Figure 7:**
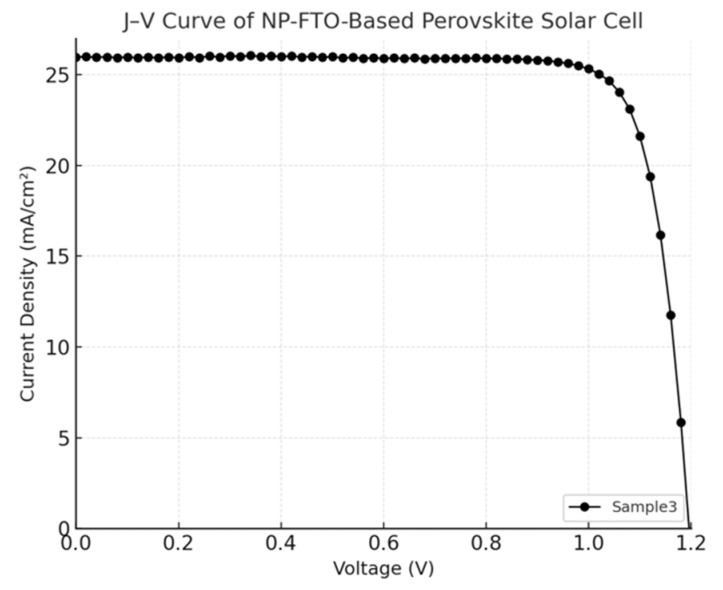
J–V curve of the NP-FTO-Sample3 device.

**Figure 8:**
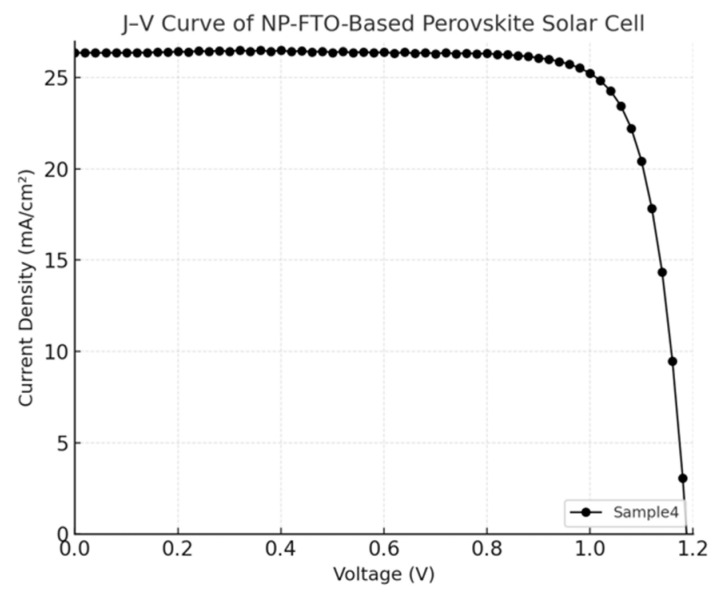
J–V curve of the NP-FTO-Sample4 device.

The current density–voltage (J–V) characteristics of perovskite solar cells (PSCs) fabricated on normal FTO and nanoplate-enhanced FTO (NP-FTO) substrates are shown in Figure 4, Figure 5, Figure 6, Figure 7 and Figure 8 Corresponding photovoltaic performance metrics are summarized in Table 1. Compared to the normal FTO device (Sample1), all NP-FTO-based devices (Samples2–5) demonstrate significant and consistent improvements in key photovoltaic parameters.

Specifically, the open-circuit voltage (Voc) increased from 1.156 V in the reference device to a peak value of 1.196 V in NP-FTO_3, likely due to suppressed trap-assisted recombination and improved interfacial band alignment at the SnO_2_/perovskite junction. The short-circuit current density (Jsc) also improved, from 24.78 mA/cm^2^ (normal FTO) to 26.35 mA/cm^2^ (NP-FTO_4), attributed to enhanced light scattering and perovskite crystallinity induced by the nanoplates.

**Figure 9:**
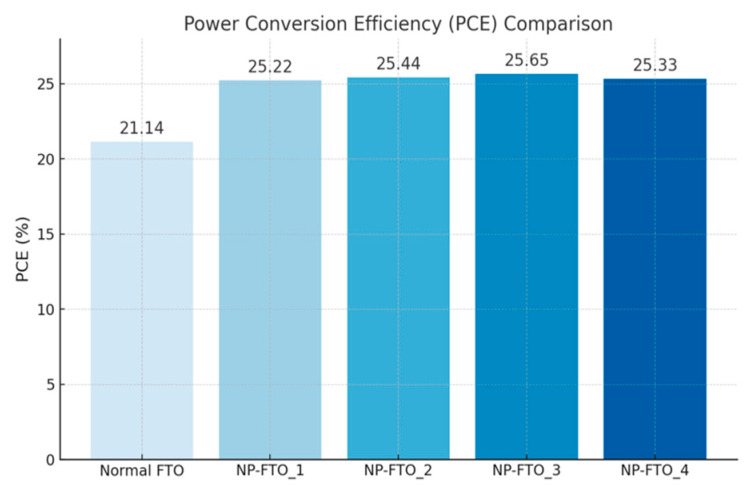
Power conversion efficiency (PCE) comparison between normal FTO and nanoplate-structured FTO (NP-FTO)-based perovskite solar cells. All NP-FTO samples show a significant improvement in PCE over the normal FTO device.

Moreover, all NP-FTO devices exhibited markedly higher fill factors (FFs), with the highest reaching 82.65% (NP-FTO_3), compared to 73.75% for the control. This suggests improved charge transport and reduced series resistance, enabled by better interfacial contact and film uniformity (Figure 9).

The resulting power conversion efficiency (PCE) increased from 21.14% in the normal FTO device to a maximum of 25.65% in NP-FTO_3. Notably, all NP-FTO samples achieved PCE values exceeding 25%, highlighting both the performance benefit and excellent reproducibility of this bottom-up nanostructured strategy.

**Figure 10:**
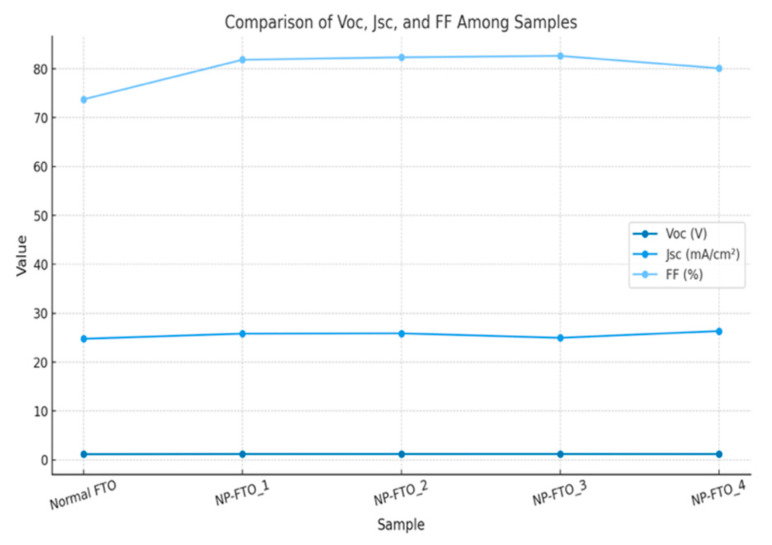
Comparison of Voc, Jsc, and FF between normal FTO and NP-FTO-based perovskite solar cells.

These findings confirm that NP-FTO substrates simultaneously promote enhanced optical, electrical, and morphological properties. By improving light management, perovskite film formation, and interfacial charge dynamics, the NP-FTO approach offers a simple yet highly effective route to boost device efficiency without altering the standard fabrication process (Figure 10).

### 3.2. Interfacial Mechanical Reinforcement Characterized by Nanoindentation

While achieving high power conversion efficiency (PCE) remains a central goal in perovskite solar cell (PSC) research, mechanical robustness is equally vital for real-world applications, especially in flexible or outdoor environments. To address this need, nanoindentation testing was employed to quantitatively assess the interfacial mechanical properties of PSCs fabricated on conventional planar FTO (normal FTO) and nanoplate-structured FTO (NP-FTO) substrates.

Five representative devices were evaluated under identical loading conditions: one based on flat FTO and four utilizing NP-FTO (denoted NP-Sample1 to NP-Sample4). The results, summarized in Table 2 and illustrated in Figure 4, reveal consistent and significant enhancements in nanomechanical performance conferred by the NP-FTO modification:

Elastic Modulus (Er): Increased from 37.22 GPa (normal FTO) to a range of 45.1–47.5 GPa across NP samples, yielding an average improvement of approximately 28.2%, indicative of enhanced interfacial stiffness and superior load-bearing capacity.

Hardness (H): Improved from 2.43 GPa to a maximum of 3.70 GPa (NP-Sample3), representing a 76.2% increase, signifying greater resistance to localized plastic deformation.

**Figure 11:**
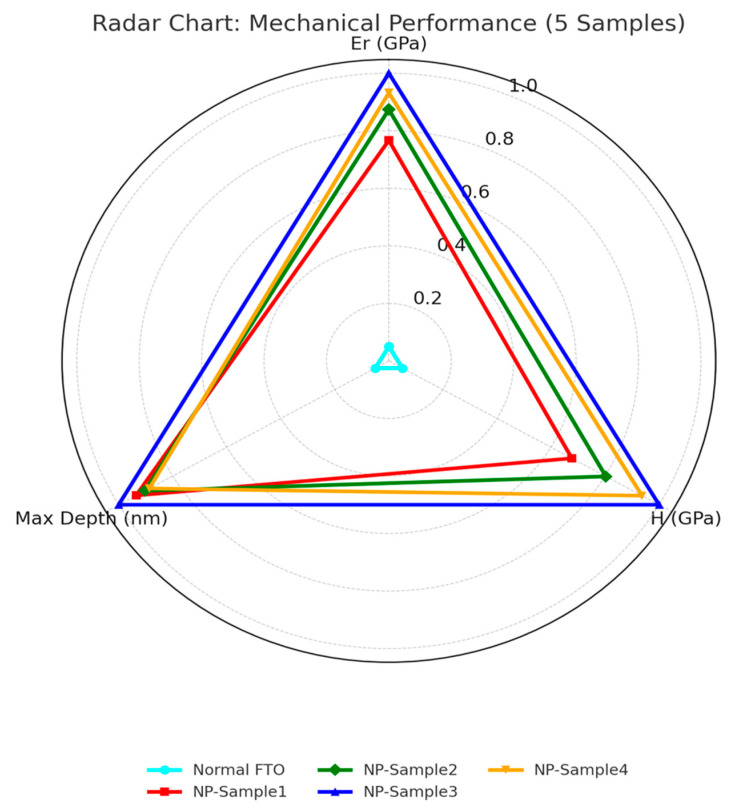
Comparison of hardness, modulus, and indentation depth in NP vs. normal FTO.

**Figure 12:**
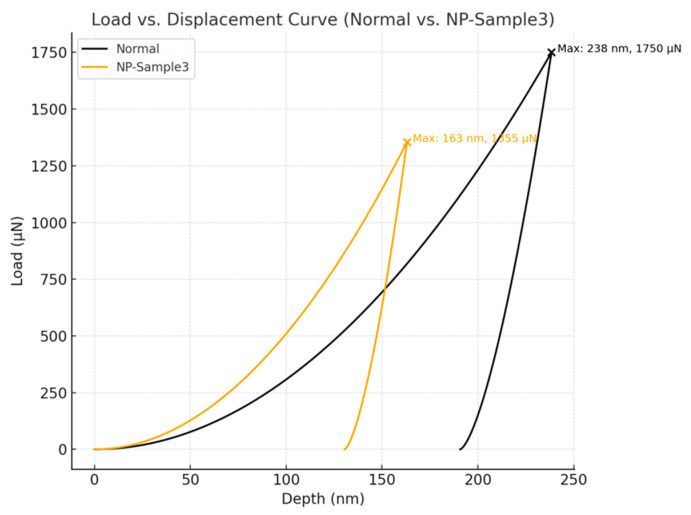
Load–displacement curves of normal FTO and NP-Sample3 measured by nanoindentation.

Maximum Indentation Depth: Reduced from 224.8 nm to as low as 163 nm, confirming a denser and more rigid interfacial structure in NP-FTO devices (Figure 11 and Figure 12).

These improvements are attributed to the vertically aligned nanoplates embedded within the FTO layer, which facilitate the formation of a three-dimensional, conformal interface between the SnO_2_ electron transport layer (ETL) and the perovskite absorber. This textured architecture offers multiple mechanical advantages:

Enhanced Interfacial Adhesion and Compactness: Nanoplates promote uniform perovskite nucleation and improved wetting on the ETL surface, resulting in dense, void-free film formation.

Stress Redistribution: The roughened surface and interpenetrating morphology allow for more uniform stress distribution across the contact area, effectively mitigating crack initiation and delamination.

Mechanical Interlocking: The embedded topography introduces anchoring effects that resist shear and tensile forces—critical for withstanding operational and environmental stresses.

Notably, these mechanical trends are strongly correlated with photovoltaic parameters, particularly the improvement in fill factor (FF) observed in NP-FTO devices. This implies a synergistic enhancement mechanism wherein interfacial reinforcement not only improves mechanical resilience but also reduces series resistance, thereby enhancing charge extraction efficiency.

From a fabrication perspective, the nanoplate FTO interface is fabricated via a bottom-up process that is compatible with low-temperature and scalable manufacturing methods, requiring no additional encapsulation or external mechanical reinforcement. This strategy thus offers a promising pathway toward high-efficiency, mechanically robust PSC modules.

In summary, nanoindentation analysis confirms that NP-FTO interfaces provide dual-functional benefits by simultaneously improving mechanical integrity and optoelectronic performance. These findings establish NP-FTO as a viable strategy for advancing the durability and commercial readiness of next-generation perovskite photovoltaics.

### 3.3. Synergistic Mechanism and Practical Implications

The remarkable dual-functional enhancement observed in NP-FTO-based perovskite solar cells stems from an integrated interfacial engineering strategy that simultaneously optimizes photon management, charge dynamics, and mechanical integrity (see Figure 3).

(a) Optical Management. Vertically aligned FTO nanoplates form a textured interface that promotes multi-angle light scattering and suppresses wide-angle reflection. Angle-resolved measurements confirm that NP-FTO increases average photon path lengths within the perovskite absorber, directly contributing to the observed Jsc improvement.
(b) Electronic Optimization. The conformal NP-FTO architecture reduces interfacial voids and trap densities, as evidenced by enhanced PLQY and increased QFLS in TRPL and TRMC studies. Improved band alignment at the NP-FTO/SnO_2_ and perovskite interface mitigates non-radiative recombination, yielding higher Voc and fill factor (FF).
(c) Mechanical Reinforcement. The three-dimensional nanoplate network provides mechanical anchoring and efficient stress redistribution under load. Nanoindentation results show a ~28% increase in reduced elastic modulus (Er) and a >70% enhancement in hardness, accompanied by a ~32% reduction in maximum indentation depth relative to planar FTO devices.

**Figure 13:**
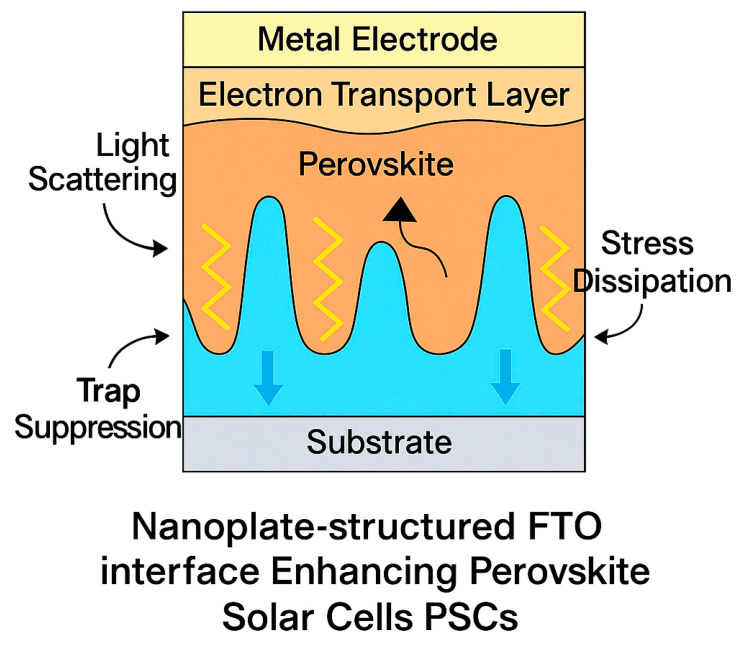
Schematic illustration of the synergistic enhancement mechanism in NP-FTO perovskite solar cells.

Collectively, these synergistic effects arise from a bottom-up NP-FTO modification that requires no additional encapsulation or external reinforcement. The fully solution-based, low-temperature fabrication process remains compatible with scalable roll-to-roll production. As a result, NP-FTO-enabled PSCs hold great promise for flexible photovoltaics, wearable electronics, and building-integrated modules, advancing the pathway toward commercial perovskite deployment (Figure 13).

## 4. Conclusions

### 4.1. Scalability and R2R Feasibility

The NP-FTO modification is implemented through a low-temperature, solution-compatible step that can be slotted before the standard SnO_2_ ETL deposition. In an R2R line, the nanoplate growth/transfer can be realized by a wet-chemistry or coating-and-etch module with drying below 200 °C, preserving the thermal budget of polymeric substrates. No additional vacuum chambers are required; the module is parallelizable to maintain line speed.

Cost/complexity: Incremental costs arise primarily from consumables and a single drying station; CAPEX and OPEX increases are limited relative to the existing TCO supply chain. We estimate a small per-area cost adder dominated by chemical usage (expected <2% of total device materials) and marginal energy overhead (<5% of front-end thermal budget).

Flexible formats: For thin-glass or polymeric TCOs, a low-aspect-ratio nanoplate geometry (<150 nm height; moderate areal density) mitigates strain concentration under bending. Design rules and in-line optical monitoring (haze/AR) can close the loop for uniformity at web scale.

### 4.2. Comparison to Other Interfacial Strategies

We benchmark NP-FTO against representative mesoporous interlayers, polymer/molecular passivation, and additive engineering.

Optoelectronic: Mesoporous layers aid charge extraction but may introduce extra roughness and thermal steps; polymer passivation lowers non-radiative loss but offers limited angular light-path gains. NP-FTO unifies light scattering and contact conformality without altering upper-stack chemistry.

Mechanical: Polymer passivation marginally affects buried stiffness; mesoporous scaffolds can improve adhesion but add porosity-related variability. NP-FTO provides a 3D mechanical interlock directly at the TCO interface, reflected in Er/H/hmax improvements aligned with FF gains.

### 4.3. Stability Outlook and Planned ISOS Tests

While this study focuses on concurrent PV–mechanical metrics, the mechanistic link—denser conformal contact and 3D anchoring—suggests better resistance to delamination, micro-crack growth, and series-resistance drift. We will include (or provide upon request) the following ISOS-aligned protocols:

1. ISOS-D-1/D-3 (ambient and damp-heat storage), 85 °C/85% RH;
2. Thermal cycling (−20 ↔ 85 °C);
3. Bending fatigue (R = 5–10 mm, 1–5 k cycles).

Key readouts: PCE retention, FF drift, Rs/Rsh, EL/PL crack mapping, and adhesion tests. We anticipate slower FF degradation and higher PCE retention for NP-FTO due to improved load distribution and reduced interfacial voids.

This work has demonstrated that engineering a nanoplate-structured FTO electrode imparts concurrent improvements in both optoelectronic performance and mechanical durability of perovskite solar cells. Compared with planar FTO controls, NP-FTO devices achieve up to a 21% relative increase in power conversion efficiency, alongside significant gains in elastic modulus and hardness. These enhancements are underpinned by (i) enhanced light harvesting through multi-angle scattering, (ii) suppressed non-radiative recombination via optimized band alignment and reduced trap densities, and (iii) robust mechanical anchoring and stress redistribution at the buried interface.

Importantly, the NP-FTO modification is realized through a scalable, low-temperature, solution-based process, requiring no additional mechanical reinforcements. This bottom-up approach is fully compatible with existing roll-to-roll manufacturing platforms and preserves device transparency and flexibility. The results offer a clear pathway for translating high-efficiency perovskite devices into flexible and building-integrated applications, bringing perovskite photovoltaics closer to commercial reality.

## 5. Future Study

Building on the demonstrated benefits of nanoplate-structured FTO (NP-FTO) in enhancing both photovoltaic performance and interfacial mechanical strength, several future research directions are worth pursuing.

Firstly, further investigation into the structural parameters of NP-FTO—such as nanoplate height, density, and crystallographic orientation—could provide a more precise understanding of their roles in light scattering, interfacial bonding, and stress dissipation. Controlled fabrication methods may help fine-tune these properties for optimal performance.

Secondly, extending the NP-FTO strategy to flexible or ultrathin substrates could open avenues for applications in wearable or portable photovoltaics. This requires adapting the synthesis process to be compatible with low-temperature, substrate-friendly conditions.

Thirdly, long-term operational stability under real-world stressors—including thermal cycling, humidity, and mechanical deformation—should be evaluated. These studies will clarify whether NP-FTO interfaces can effectively suppress delamination and fatigue-related failure modes over extended lifetimes.

Finally, integrating NP-FTO with additional interfacial engineering strategies, may further enhance device efficiency and durability. Exploring these synergistic effects could enable new design paradigms for robust, high-performance PSCs.

## Figures and Tables

**Table 1:** Comparison of photovoltaic parameters between normal FTO and NP-FTO-based perovskite solar cells.

Sample	Voc (V)	Jsc (mA/c^2^)	FF (%)	PCE (%)
Normal FTO	1.156	24.78	73.75	21.14
NP-FTO-Sample1	1.193	25.82	81.85	25.22
NP-FTO-Sample2	1.194	25.88	82.36	25.44
NP-FTO-Sample3	1.196	24.97	82.65	25.65
NP-FTO-Sample4	1.187	26.35	80.10	25.33

**Table 2:** Statistical summary of nanomechanical properties in normal FTO and NP-modified devices.

Sample	Er (GPa)	H (GPa)	Max Indentation Depth (nm)
Normal FTO	37.22 ± 1.02	2.43 ± 0.24	224.80 ± 12.83
NP-Sample1	45.1 ± 2.03	3.29 ± 0.13	167 ± 11.58
NP-Sample2	46.2 ± 1.92	3.45 ± 0.18	169 ± 11.6
NP-Sample3	47.5 ± 1.66	3.70 ± 0.12	163 ± 10.3
NP-Sample4	46.8 ± 1.98	3.62 ± 0.17	170 ± 10.92

## Data Availability

If there are any other requirements, anyone can request them from the corresponding author.
